# Optimizing military mental health and stress resilience training through the lens of trainee preferences: A conjoint analysis approach

**DOI:** 10.1080/08995605.2024.2324647

**Published:** 2024-03-14

**Authors:** Callista A. Forchuk, Ilyana Kocha, Joshua A. Granek, Kylie S. Dempster, William A. Younger, Dominic Gargala, Rachel A. Plouffe, Suzanne Bailey, Kim Guest, J. Don Richardson, Anthony Nazarov

**Affiliations:** a MacDonald Franklin Operational Stress Injury Research Centre, Lawson Health Research Institute, London, Ontario, Canada; b Defence Research and Development Canada (DRDC) – Toronto, Department of National Defence, Ottawa, Ontario, Canada; c Department of Psychiatry, Schulich School of Medicine & Dentistry, Western University, London, Ontario, Canada; d Canadian Forces Health Services Group, Canadian Armed Forces, Ottawa, Ontario, Canada; e Department of Psychiatry and Behavioral Neurosciences, McMaster University, Hamilton, Ontario, Canada; f St. Joseph’s Operational Stress Injury Clinic, St. Joseph’s Health Care London, London, Ontario, Canada

**Keywords:** Military, resilience training, mental health, stress resilience

## Abstract

Effective mental health and stress resilience (MHSR) training is essential in military populations given their exposure to operational stressors. The scarcity of empirical evidence supporting the benefits of these programs emphasizes the need for research dedicated to program optimization. This paper aims to identify the relative importance of MHSR training attributes preferred by military members. Conjoint analysis (CA), an experimental method used to prioritize end-user preferences for product feature development, was conducted using an online survey with 567 Canadian Armed Forces (CAF) personnel. Participants made a series of choices between hypothetical MHSR training options that were systematically varied across seven training attributes. Each training attribute consisted of 3–4 variations in the nature of the attribute or its intensity. Participants also completed questions on health beliefs, mental health and previous MHSR training experiences, and demographics, to assess whether preferences varied by individual characteristics. CA demonstrated that instructor type, leadership buy-in, degree of skills practice, and content relevance/applicability were attributes of highest and relatively equal importance. This was followed by degree of accessible supplemental content. Lowest importance was placed on degree of behavioral nudging and demographic similarity between the trainee and trainer. Sociodemographic factors were not associated with MHSR training preferences. Programs that incorporate expert-led instruction, demonstrate leadership buy-in, embed practical applications within simulated stress environments, and provide a digitally-accessible platform to augment training may be well-received among military members. Understanding and accommodating personal preferences when designing MHSR training programs may increase relevance, foster acceptance and trust, and support sustained engagement.

**What is the public significance of this article?—**This study suggests that military personnel prioritize mental health and resilience training programs in which there is instructor expertise in mental health, leadership buy-in, and a high degree of skill practice and content relevance. Personnel were also interested in having easy access to training content, but were less concerned with having prompts or reminders to practice training skills or with having a demographic match between trainers and trainees.

Mental health and stress resilience (MHSR) have become essential components within many military organizations, with the goal of equipping members to handle operational stressors. These programs typically focus on promoting long-term psychological health and short-term performance by enhancing mental health literacy, teaching stress management skills, and changing negative attitudes toward help-seeking (Bailey, 2015). Various programs, such as the American Force Health Protection and Readiness program (Bowles & Bates, 2010), the British Trauma Risk Management program (Greenberg et al., 2008), the Australian Arts for Recovery, Resilience, Teamwork, and Skills program (Temby et al., 2015), and the Canadian Road to Mental Readiness (R2MR) program (Bailey, 2015), are implemented globally. Despite the widespread use, literature on their effectiveness has yielded mixed results, with studies showing positive effects (e.g., lower levels of stigma), no effects, or even detrimental effects (e.g., decreased willingness to engage in help-seeking behaviors; Adler et al., 2009; Cacioppo et al., 2015; Crane et al., 2019; Falon et al., 2020; Fikretoglu et al., 2019; McInerney et al., 2022; Saltzman et al., 2011; Sharpley et al., 2008). These inconsistent findings can be attributed to logistical challenges of running large-scale trials, the multifaceted nature of MHSR programs, the variation in key ingredients of the interventions across programs, lack of consideration for fidelity, vague program logic models, poor measurement of intended program outcomes, and selection of study methodologies that do not allow for a high degree of confidence in the findings. The lack of demonstrated benefit could, in part, be due to a lack of program adherence and engagement among trainees. Indeed, trainee engagement is a strong predictor of outcome of a range of clinical and non-clinical interventions (Elkin, 1999; Kwan et al., 2010), highlighting the necessity of aligning program components with trainee preferences. By understanding and accommodating these preferences, individuals are likely to become more engaged. This heightened engagement can foster greater commitment to the program, thereby enhancing the likelihood of achieving the intended outcomes. This project aimed to extract the prioritized features of MHSR training intended for a military audience.

The importance of trainee needs and preferences is reflected among the fundamental principles of MHSR training, which include “relevant purpose and content” and “user acceptability” (Castro & Adler, 2011). These principles highlight the necessity of user preferences in designing effective programming, as *relevance* requires an understanding of trainee needs to ensure content is appropriately matched, and *user acceptability* requires an understanding of trainee preferences to ensure training feels useful and satisfactory. In the related field of mental health treatments, meta-analytic findings indicate that clients matched to their preferred interventions, or who were involved in treatment selection, experienced greater mental health benefits, and were more satisfied and likely to adhere to treatments (Delevry & Le, 2019; Lindhiem et al., 2014). It is plausible, for example, that military members may wish to see a greater proportion of MHSR content focused on practical examples, more opportunities to practice skills in realistic environments, and see evidence of military leadership support for the program (Nazarov et al., in press). Overall, research shows that extracting user preferences is crucial for guiding the implementation of mental health-related interventions.

Due to the multifaceted nature of MHSR programs and limited resources related to program design and execution, an extraction of user preferences will need to consider practical trade-offs, opportunity cost, and the relative importance of program features. Techniques from marketing research, such as conjoint analysis (CA), have become increasingly popular in health research for their ability to derive these relative preferences (Larsen et al., 2021). In CA, participants are typically presented with a series of choices between two or more product options (e.g., training programs) that vary on pre-specified product “attributes” (e.g., instructor qualities) across a realistic range of possibilities, or “attribute levels” (e.g., instructor is a uniformed peer). Relative preferences can then be statistically derived and used to guide realistic program recommendations. As individuals themselves are multifaceted, user characteristics should be considered when determining preferences, as demographic variables such as age and gender have been predictive of unique health care preferences (Gould, 2011; Jung et al., 2003). More generally, individuals may differ in predictable patterned ways, such that there may be clusters of individuals who share similar preferences, as has been found in previous mental health research employing CA (e.g., Cunningham et al., 2017).

The objective of this study was to identify the relative importance of MHSR training features among a sample of active military personnel. Specifically, we aimed to 1) identify the relative importance of MHSR training features according to military personnel, 2) discern patterns in participant preferences and understand their distinctions based on participant characteristics, and 3) suggest strategies to effectively implement the identified improvements. Results of this study can be used to inform priorities for enhancing the design and implementation of MHSR programs.

## Method

### Participants and recruitment

A total sample of 567 Canadian Armed Forces (CAF) personnel participants, comprising both French-speaking and English-speaking individuals, was recruited via e-mail invitations sent through the secure internal electronic network used by DND and CAF. An e-mail list was created with stratification based on rank (i.e., Junior Non-Commissioned Member [NCM], Senior Non-Commissioned Officer [NCO], Junior Officer, and Senior Officer) and component type (i.e., Regular Force or Reserve Force). A target sample size of 400 was chosen based on recommendations for CA of at least 300 participants (Orme, 2006). A total of 4155 invitations were distributed, assuming a response rate of 20% and a survey completion rate of 65%. These estimates were derived from previous studies involving CAF participants (Forchuk et al., 2022; Granek et al., 2020). Participants could choose to complete the survey in French or English. The current analysis focused on 508 English-speaking participants; French-speaking participants were not included in the present analyses due to limitations of the CA platform.

### Attribute development and survey design

Attributes and levels were derived from (a) the qualitative synthesis of training preference feedback, obtained from CAF member participants (*n* = 343–503) of a previous Defence Research and Development Canada (DRDC)-led study on MHSR training optimization in the CAF, (b) research relating to resilience training, the MHSR program, and educational or mental health treatment preferences, and (c) consultation with MHSR program developers.

Attributes were selected based on their relevance, modifiability, and independence from one another, following consensus guidelines for CA in health research (Bridges et al., 2011; Lancsar & Louviere, 2008). Initial attributes were selected to reflect the range of themes identified in a previously-conducted qualitative synthesis (Nazarov et al., in press), with emphasis placed on those that were most frequently mentioned (e.g., realistic training). Attributes were revised in collaboration with experts in military research and survey design, and with MHSR program developers to represent a realistic range of options, and to conform to CA best practices, which recommend a maximum of 12 attributes with approximately 3–4 levels each, and with an approximately equal number of levels across each attribute (Bridges et al., 2011; Lancsar & Louviere, 2008). Final revisions were made based on pilot-testing by the authors. As a result, a total of seven unique attributes were included. To alleviate cognitive load associated with the simultaneous comparison of training options across 7 attributes, attributes were split across two different surveys (Survey A and Survey B), with Survey A containing attributes related to the delivery and content of the course, and Survey B containing attributes related to the leadership support and reinforcement of course material. One attribute (i.e., degree of realistic/practical training) was included in both surveys as an anchor to be able to compare relative preferences between surveys. Participants were randomized between the two survey versions. All attributes were described by 3–4 levels and distributed to participants using a full profile design (i.e., each option included one level from each attribute) across 10 forced-choice tasks with two options presented at a time. A complete list of attributes and levels is presented in Table 1. Participants were asked supplementary open-ended questions following the CA module about missed attributes, changes in preferences over their careers, and the integration of training into a smartphone application; analysis of these responses is outside of the scope of this paper.

**Table 1.** Attributes and Levels for Conjoint Analysis.

Attribute Content	Levels
**Content Relevance/Applicability** *(Surveys A and B)*	*Realistic examples/applications are incorporated*: never throughout the training throughout some of the training throughout most of the training throughout all of the training
**Instructor Type** *(Survey A)*	*R2MR instructors are*: uniformed leaders uniformed peers civilian mental health and resilience experts uniformed mental health and resilience experts
**Skill Practice** *(Survey A)*	*Training is provided*: without opportunity to practice skills with opportunity to practice skills without simulated stress with opportunity to practice skills under simulated stress
**Demographic Similarity Trainee/Trainer** *(Survey A)*	*Training instructors/video actors represent my personal characteristics (e.g., gender, ethnicity, religion)*: none of the time some of the time most of the time all of the time
**Supplemental Content** *(Survey B)*	*Supplemental content is provided through*: handouts and e-mail a digital portal only accessible on DND network or devices a digital portal accessible from anywhere and on personal devices
**Leadership Buy-In** *(Survey B)*	*Leaders*: do not actively support/model training promote training model skills outside of R2MR context provide opportunities to practice R2MR
**Nudging** *(Survey B)*	*Messaging to reinforce skill practice is through*: a personalized app tailored to your performance and well-being generic emails sent through app or e-mail posters mounted in common areas

Participants were exposed to training options comprising the levels of all four attributes in their respective survey version. Each attribute began with the same stem, with only the ending differing according to the specific level. R2MR = Road to Mental Readiness.

### Demographics, mental health, and MHSR experiences

Participants were asked their gender, age, ethnicity, education, and military component, element, and rank. Participants were asked whether they experienced a problem related to stress, emotions, alcohol, or anything else affecting their well-being (1 item; yes/no), and whether they have professional or educational experience related to mental health (1 item; yes/no). Prior exposure and perceptions of MHSR were gathered using four items which examined whether (yes/no), when (i.e., months ago), and for which purpose (i.e., training context) MHSR training was personally experienced.

### Health locus of control

Health locus of control was gathered using an adapted version of the Multidimensional Health Locus of Control (MHLC) Scale, a 19-item scale that asks participants to rate their agreement from 1 (strongly disagree) to 6 (strongly agree) on items assessing health locus of control. The items were adapted by the authors to relate to mental rather than physical health.

### Procedure

**Figure 1.**
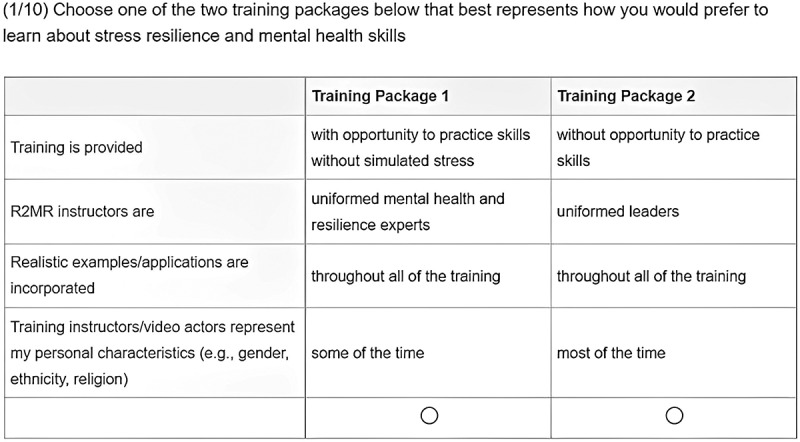
A sample of the format used in the conjoint analysis 10 choice tasks completed by each participant.

The study protocol was approved by the Health Research Ethics Committee of DRDC. Data was collected using the CA module of the web-based platform Qualtrics. Participants were randomly allocated to either Survey A or B, which differed only in the CA attributes presented, with all other portions of the survey held constant. Qualtrics automatically generated unique choice configurations for each participant, which were balanced in the representation of levels and attributes within and between participants. For CA choices, participants were asked to imagine they were a CAF member restarting their career, with full control to create their ideal MHSR training. Participants were then asked to select their preference among two options which each described the levels of four attributes (Figure 1); this was repeated across 10 trials. The data from this study are property of DRDC and are not allowed to be shared publicly.

### Data analysis

Qualtrics was used to analyze the CA survey data. Qualtrics uses Hierarchical Bayes estimation to derive individual-level preferences in the form of part-worth utilities, which reflect the preference for or “utility” of each attribute level based on participants’ choice responses. The average utility score of each level is also calculated for the sample to reflect the ordinal ranking of attribute levels. The part-worth utilities are zero-centered and generally range from −5 to +5. These utilities are used to derive attribute importance metrics by taking the distance between the best and worst level within an attribute, where larger distances (i.e., more preference variability within the attribute) reflect greater attribute importance. Attribute importance scores are ratio-scaled from 0 to 1 (i.e., relative proportion to other attributes included in the survey; e.g., attribute with an importance score of .2 is twice as important as an attribute with an importance score of .1). R Version 4.2.2 (R Core Team, 2021) was used to perform additional analysis, including cluster analysis and the calculation of descriptive statistics. Pearson chi-square tests were used to compare participants between surveys on sociodemographic and military characteristics.

As part of an exploratory analysis intended to identify participants who shared similar preference patterns according to their part-worth utility scores, cluster analysis using *k-*means clustering was implemented to confirm clusters obtained through Qualtrics. The optimal number of clusters was derived separately for Survey A and B using the *silhouette* function from the *cluster* package in R. Silhouette coefficients range from −1 to +1, where −1 suggests the clusters are dissimilar or misclassified, and +1 suggests high within-cluster similarity and that clusters are well-differentiated from one another (Rousseeuw, 1987). Each survey dataset was then partitioned into its optimal number of clusters, and participants of different clusters were compared using chi-square tests for categorical variables and ANOVA tests for continuous variables to determine whether sociodemographic and military variables could predict cluster membership. Utility scores within clusters were also inspected to characterize their distinct preference patterns.

## Results

### Participants

Participant characteristics are summarized in Table 2. Participants were primarily white men between 25 and 54 years old, who had completed high school or university. Participants were primarily Regular Force, in the Army, and junior noncommissioned members (NCM). Chi-square tests revealed no differences in military or sociodemographic variables between participants in Survey A and B (Table 2).

**Table 2.** Participant Characteristics.

	*n* (%)			
Variable	Combined (*n* = 508)	Survey A (*n* = 261)	Survey B (*n* = 247)	*p-*value
*Gender*				.68
Man	356 (70.1)	184 (76.3)	172 (72.9)	
Woman	112 (22.1)	54 (22.4)	58 (24.6)	
Other	3 (0.6)	1 (0.4)	2 (0.8)	
Prefer not to say	6 (1.2)	2 (0.8)	4 (1.7)	
*Age*				.28
Younger than 25	40 (7.9)	24 (10.0)	16 (6.8)	
25 to 34	139 (27.4)	74 (30.7)	65 (27.5)	
35 to 44	153 (30.1)	80 (33.2)	73 (30.9)	
45 to 54	120 (23.6)	53 (22.0)	67 (28.4)	
Older than 55	25 (4.9)	10 (4.1)	15 (6.4)	
*Ethnoracial background*				.67
White/Caucasian	379 (74.6)	191 (73.2)	188 (76.1)	
East and Southeast Asian	38 (7.5)	18 (6.9)	20 (8.1)	
Black/African/Caribbean	19 (3.7)	9 (3.4)	10 (4.0)	
Indigenous Peoples (e.g., First Nations, Inuit, Metis, etc.)	16 (3.1)	5 (1.9)	11 (4.4)	
Latin American/Hispanic	10 (2.0)	6 (2.3)	4 (1.6)	
South Asian (e.g., East Indian, Pakistani, Sri Lankan, etc.)	8 (1.6)	4 (1.5)	4 (1.6)	
West Asian (e.g., Afghan, Iranian, Arab, etc.)	5 (1.0)	4 (1.5)	1 (0.4)	
Other (please specify)	11 (2.2)	7 (2.7)	4 (1.6)	
Prefer not to say	10 (2.0)	6 (2.5)	4 (1.7)	
*Education*				.97
Some secondary school	18 (3.5)	8 (3.3)	10 (4.3)	
Completed secondary school	99 (19.5)	49 (20.4)	50 (21.4)	
Some college/CEGEP	55 (10.8)	30 (12.5)	25 (10.7)	
Completed college/CEGEP	65 (12.8)	33 (13.8)	32 (13.7)	
Some university (undergraduate)	59 (11.6)	28 (11.7)	31 (13.2)	
Completed university (undergraduate)	120 (23.6)	64 (26.7)	56 (23.9)	
Graduate or professional degree	58 (11.4)	28 (11.7)	30 (12.8)	
*Military component*				.67
Regular Force	405 (79.7)	203 (84.6)	202 (86.0)	
Reserve Force	70 (13.8)	37 (15.4)	33 (14.0)	
*Military element*				.42
Navy	73 (14.4)	37 (15.4)	36 (15.2)	
Army	247 (48.6)	131 (54.4)	116 (48.9)	
Air Force	152 (29.9)	69 (28.6)	83 (35.0)	
Other	6 (1.2)	4 (1.7)	2 (0.8)	
*Military rank*				.53
Junior NCM	197 (38.8)	99 (41.2)	98 (41.5)	
Senior NCM	114 (22.4)	56 (23.3)	58 (24.6)	
Junior Officer	94 (18.5)	53 (22.1)	41 (17.4)	
Senior Officer	71 (14.0)	32 (13.3)	39 (16.5)	

NCM = non-commissioned member. *P*-values correspond to Pearson chi-square tests of independence.

### Importance scores

**Figure 2.**
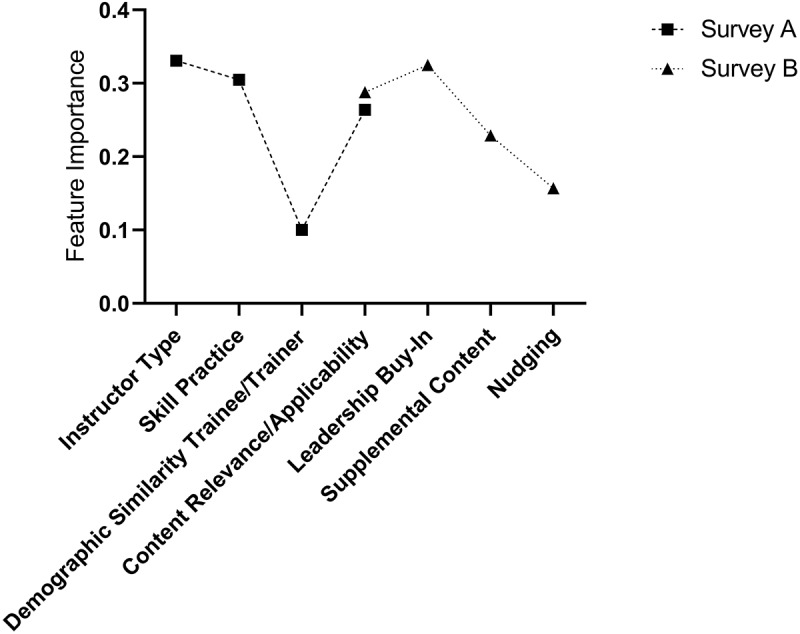
Representation of attribute importance scores.

Importance scores are visualized in Figure 2. The importance score of the content relevance/applicability attribute that was common across the two surveys was relatively equal across versions, allowing for a cross-comparison of importance scores ranking across survey versions. Overall, the instructor type (Survey A; 0.35), leadership buy-in (Survey B; 0.32), and degree of skills practice (Survey A; 0.30) attributes were most influential and of relatively equal importance, along with the content relevance/applicability attribute (Survey A; 0.26, Survey B; 0.29) shared across both surveys. This was followed by degree of accessible supplemental content (Survey B; 0.23). The attributes with the lowest importance scores were degree of behavioral nudging (Survey B; 0.16) and demographic similarity between the trainee and trainer (Survey A; 0.09).

### Utility scores

**Figure 3.**
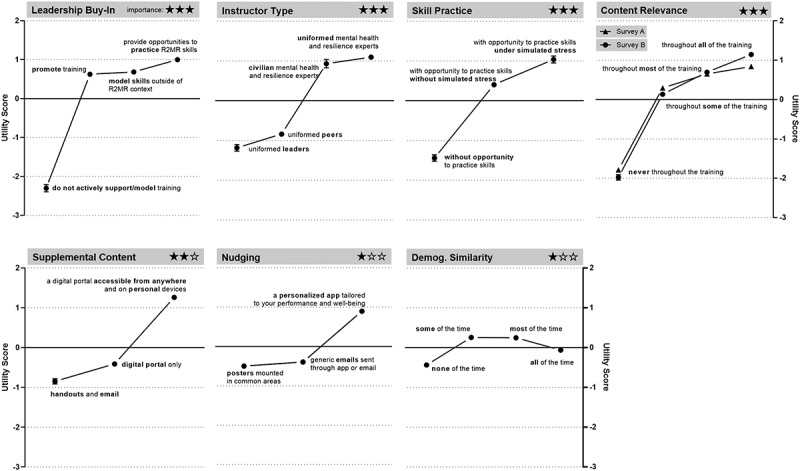
Average utility scores of attribute levels. Stars are used to indicate broad estimates of relative importance scores derived from Figure 2.

Average utility scores for both surveys are presented in Figure 3 (see Table S1 for exact values). Participants preferred the opportunity to practice skills learned in their MHSR training. There was a tendency to favor practice while under simulated stress, but practice without simulated stress was still preferred over no practice. Participants preferred uniformed or civilian MHSR experts as instructors, rather than uniformed peers or leaders. Some degree of personal representation was preferred, with equal utility scores for “some of the time” and “most of the time.,” although the magnitude of the utility scores were low.

Participants favored accessible supplemental content, with the highest estimated utility for content to be available through “a digital portal accessible from anywhere and on personal devices.” Utility scores indicated a minimal advantage for a digital portal only accessible on DND network or devices over handouts and e-mail. Some degree of leadership support emerged as preferable over none, with similar estimated utility scores for leaders promoting training and modeling skills outside of an MHSR context. Participants most preferred that leaders offered practice opportunities for MHSR skills. However, the distinctions between specific types of leadership support (e.g., practice opportunities vs. training promotion) were relatively small, as long as some support was offered. Both surveys revealed that participants preferred any incorporation of realistic or practical applications, with minimal differentiation between degrees of incorporation. Lastly, nudging held the least influence on participant choices, but a personalized app was the most preferred method of cueing participants to practice their skills.

### Cluster analysis

The highest average silhouette for the delivery and content attributes survey (Survey A) was found at *k* = 3 clusters (approximately 0.28), and *k* = 2 for practical support and reinforcement of course material survey (Survey B) (approximately 0.36).

**Figure 4.**
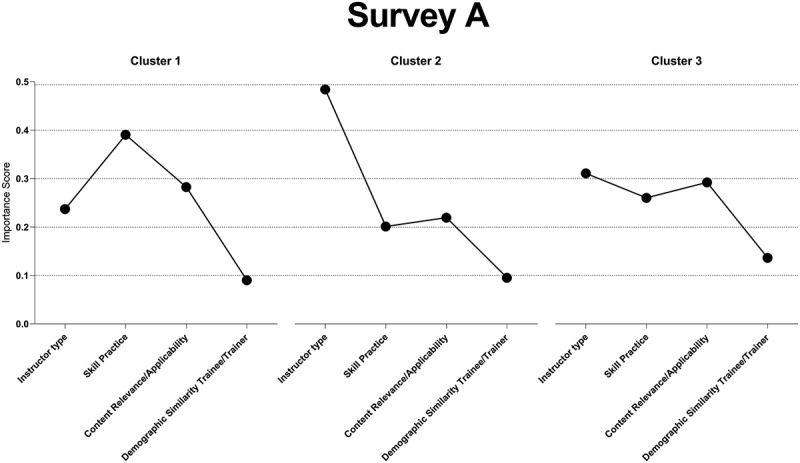
Importance scores for Survey a clusters.

The delivery and content attributes survey (Survey A) emphasized the degree of skills practice with an importance score of 0.39, content relevance/applicability at 0.28, instructor type at 0.24, and degree of demographic similarity between the trainee and trainer at 0.09 in Cluster 1. Cluster 2 placed a higher weight on instructor type at 0.48, followed by content relevance/applicability at 0.22, degree of skills practice at 0.20, and degree of demographic similarity at 0.09. Finally, Cluster 3 assigned relatively balanced importance to instructor type at 0.31, content relevance/applicability at 0.29, and degree of skills practice at 0.26, while the degree of demographic similarity between trainee and trainer had a score of 0.14 (see Figure 4).

**Figure 5.**
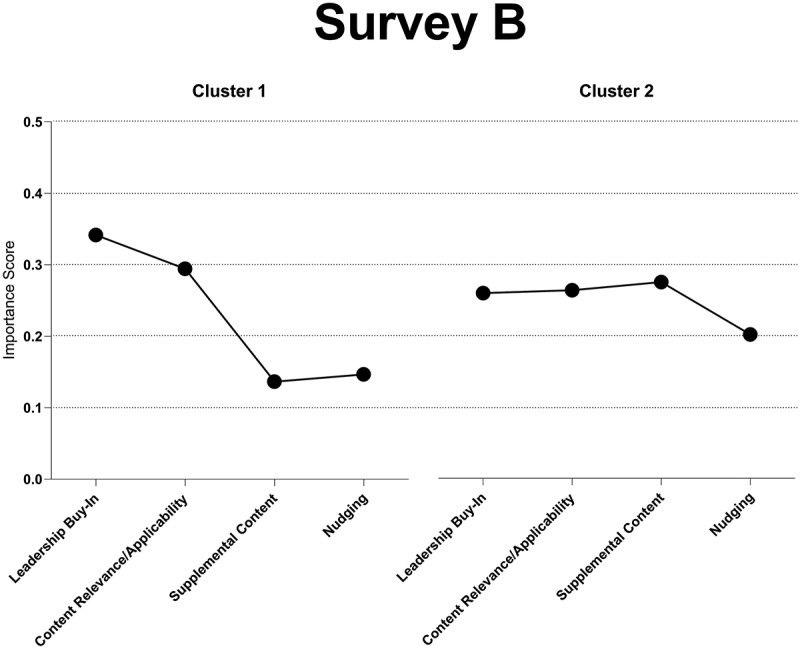
Importance scores for Survey B clusters.

The practical support and reinforcement of course material survey (Survey B) prioritized leadership buy-in with an importance score of 0.34, followed by content relevance/applicability at 0.29, degree of behavioral nudging at 0.15, and degree of accessible supplemental content at 0.14 in Cluster 1. Cluster 2, on the other hand, emphasized the degree of accessible supplemental content at 0.27, closely followed by content relevance/applicability at 0.26, leadership buy-in at 0.25, and degree of behavioral nudging at 0.20 (see Figure 5).

Although the current analysis revealed statistically significant clusters, it did not reveal statistically significant associations between the demographic characteristics of participants and the clusters in either survey. Descriptive statistics by cluster for demographics, R2MR exposure, mental health history, and health locus of control are presented in Table S2 and Table S3 for Surveys A and B, respectively.

## Discussion

This is the first study to utilize CA to systematically explore and prioritize the preferences of military personnel concerning their perceptions of MHSR training. The findings highlight key opportunities to refine MHSR training according to the preferences of a military population. The significant emphasis on leadership support, qualities of MHSR instructors, and realistic scenarios provides a nuanced understanding of what resonates with military personnel.

The significance placed on leadership support by the participants suggests that focusing on this aspect could substantially improve military member satisfaction with the program. Even modest endorsements or visible support from military leaders for MHSR training can have positive effects on the perception of these programs among military personnel. The hierarchical structure of military organizations gives leaders substantial control and influence. Through support and modeling, leaders can influence how subordinates interpret their experiences and engage with MHSR material (Bartone, 2006). This becomes especially important during exposure to highly demanding and stressful environments (Hannah et al., 2010). Conversely, an absence of such support may foster negative views of the training, hindering its potential impact. Mental health stigma can be a barrier to effective MHSR training with self-stigmatization potentially deterring individuals from fully engaging with course material (Adler, 2013). Leaders framing MHSR training around performance and ensuring opportunities for immediate skill transfer post-training can enhance program effectiveness (Crane et al., 2022). These observations are consistent with existing research highlighting the presence of mental health stigma within the military (e.g., Hoge et al., 2004) and the emphasis on the need for active leadership engagement to counteract this stigma (Greene-Shortridge et al., 2007).

Military personnel clearly expressed a strong preference for MHSR instructors who possess expertise in the field, and this preference remained consistent whether the instructors were uniformed or civilian experts. Accordingly, it is advisable that instructors chosen for these programs are not only highly qualified in MHSR but also capable of articulating their expertise in a manner that resonates with and gains the confidence of their trainees. Although the survey did not delve into the specifics of what constitutes such expertise (e.g., distinctions between educational qualifications or professional experiences), it is evident that establishing credibility through tangible professional experiences or recognized educational credentials could significantly enhance the reception and perceived value of the training among military members. In contrast, peers or leaders whose expertise is not clearly demonstrated or articulated may find their instruction less favorably received. This not only influences the necessity for improving program fidelity through specialized instructor training, as outlined by Bailey and Guest (2022), but also underscores the need to effectively convey the stringent selection process and rigor of the instructor training to participants.

Realistic scenarios and practical applications emerged as important factors when determining MHSR preferences across both surveys. Allocating more time for MHSR training with scenarios and skills that are personally applicable may improve satisfaction with the offered training. For instance, devising team-building exercises that mirror actual military operations, could make the training more engaging and relatable. Providing opportunities to apply learned strategies in contexts similar to military deployments might also reinforce the skills being taught. The results parallel those related to leadership support, suggesting that even modest enhancements in the incorporation of realistic examples could yield substantial improvements in the satisfaction of military members with their training. Similarly, simulated stress practice was also highlighted as a key preference. This could involve alternative engagement methods and immersive experiences, such as realistic field exercises and digitally simulated environments (e.g., virtual reality), which have been investigated for use in military populations for MHSR training with promising results (Mjelde et al., 2016; Pallavicini et al., 2016). Program developers have acknowledged this need for enhanced training environments. For example, the R2MR program has broadened its array of scenarios and activities designed to mimic stress-inducing conditions in the classroom (S. Bailey, personal communication, September 13, 2023). This approach allows participants to explore how heightened activation levels can affect their cognitive load management, team interactions, communication skills, and decision-making processes. Activities are deliberately chosen to mirror real-world stressors participants may encounter, such as making decisions under pressure, coordinating tasks in chaotic situations, and working with incomplete or ambiguous information. Furthermore, collaboration with leadership allows to integrate this practice into broader training contexts. This integration aims to establish habits and routines that incorporate mental skills as a standard component of daily tasks.

Emerging technologies offer a promising opportunity to implement personalized scenarios for resilience training at scale. Recent studies provide evidence to underline the effectiveness of stress management training in enhancing overall performance in critical simulated situations (Sigwalt et al., 2020). A robust and adaptable framework for monitoring and refining the realism and relevance of scenarios within training is instrumental in fostering trainee engagement and uptake. Solely relying on field practice for training under stress might not be viable from a financial and logistical standpoint. When employing classroom or off-site training under scenario paradigms, it’s essential to maintain optimal levels of immersion to guarantee trainee involvement and interest. Future research could investigate the most efficient modalities and immersion levels necessary to tangibly impact participants’ stress arousal. Personalized strategies, such as biofeedback, might offer valuable insights into tailoring these approaches.

Finally, other factors were less of a priority for military personnel – these included access to complementary content, utilization of behavioral nudging, and attention paid toward aligning the personal characteristics of the trainer with the trainee. Using complementary content to foster discussions and highlight key course concepts, rather than repetitive lecturing, can enhance understanding of challenging material (Price et al., 2012). It should be noted that the lack of explicit preference for behavioral nudging does not negate its potential effectiveness as a strategy for behavioral change (Iversen et al., 2021; Milkman et al., 2021; Orbell & Verplanken, 2010). Its effectiveness often lies in its subtlety to influence decisions without overt persuasion or coercion (Marchiori et al., 2017). The absence of demand for behavioral nudging in our study might reflect a lack of familiarity with the concept or how it could be applied within the context of MHSR training. Alternatively, it may indicate that respondents were focused on more immediate and tangible aspects of training. As such, developers of MHSR programs should not necessarily dismiss the potential utility of behavioral nudging and instead, careful consideration should be given to how it might be strategically integrated into the training, aligned with the broader goals and values of the program, and communicated in a way that resonates with military personnel.

Our study uncovered discernable patterns within the preferences for MHSR training, revealing that individuals exhibited unique preferences that allowed them to be grouped into distinct clusters based on their responses to the CA. These clusters reflect different priorities and values associated with MHSR training, emphasizing the multifaceted nature of the preferences within the military population. Interestingly, the sociodemographic, military, and personal variables collected during the study did not correlate with these clusters, meaning that the traditional markers like age, rank, gender, and element were not predictive of specific training preferences, nor were basic personal experiences such as past MH help-seeking, health locus of control, or professional training. This absence of association suggests a more complex interplay of factors influencing preferences, transcending surface-level demographic categorizations; this warrants a consideration of other unexplored variables that might have a greater predictive value. For instance, the individual’s more nuanced personal experiences, perceived needs, attitudes toward mental health, and even unique physiological responses to stressors might play a more important role in shaping these preferences. Additionally, it is important to consider the role of multilevel influences, as personnel are nested within specific training and occupational environments, and guided by particular leadership styles. Future research incorporating multilevel analysis could examine how between-person preferences vary according to such higher-order factors, as well as how personal preferences evolve over time in response to career advancements and accumulation of experience. The findings hint at the rich complexity of the factors that may influence how military personnel value different elements of MHSR training. Future research aimed at identifying these factors may provide more targeted insights for tailoring MHSR training programs to meet the diverse and specific needs of the military population, potentially enhancing both engagement and effectiveness.

Although the study design implemented here simulated real-world decisions that considered competing needs and values for MHSR training (McCullough, 2002) and offered an experimental approach less biased than rating scales (Cunningham et al., 2010), it is not without limitations. The number of attributes examined during the CA was limited to seven to reduce participant fatigue and misinterpretation, potentially excluding critical attributes from the analysis. The division of these attributes into two surveys was similarly necessary to prevent participant fatigue and cognitive overload. This approach introduced a challenge in comparing all attributes directly across the entire sample. We sought to mitigate this limitation by including an “anchor” attribute in each survey, allowing for a form of linkage between the two sets of responses. Each survey consisted of different individuals, and thus it was not possible to determine the extent to which relative preferences might have varied between samples, although participants were similar in terms of sociodemographic and military composition between surveys. Future research on trainee preferences should consider methods which allow comparisons between components of training delivery, content, external support, and learning reinforcements; for example, partial profile CA designs could be used, as these allow for presentation of a subset of attributes in each choice to mitigate risks of participant fatigue and cognitive overload (Bridges et al., 2011). Moreover, it is crucial to recognize that preferences expressed by participants are shaped by a host of individual and contextual factors, and what is favored by participants may not be perfect indicators of the actual or intended effectiveness of the MHSR program. Future research should consider ongoing, continuous monitoring of participant preferences, as well as the relations of such preferences with relevant outcomes, such as program effectiveness and trainee engagement. This would allow for real-time adjustments to the MHSR program, enabling it to evolve in response to the changing needs and preferences of military personnel. Further, given the distinctions found between facets of MHSR training, research which carefully selects and documents MHSR training characteristics, such as instructor expertise, along with participant perceptions of the training content and external support and degree of content relevance, would improve our ability to adapt programs with greater precision.

This study represents a unique, quantitative approach toward understanding the diverse and multifaceted preferences of military personnel with regard to MHSR training. By identifying the relative importance of attributes such as leadership support, instructor expertise, and realistic scenarios, along with their corresponding magnitude and types, it offers insights that are instrumental in customizing and prioritizing future developments of MHSR programs. These findings underscore the value of considering individual preferences and their relative importance in the design and implementation of effective MHSR programs, thereby enhancing their resonance and potential impact within the target audience.

## Supplementary Material

Table S1. Average Utility Scores of Survey A and Survey B.docx

Table S2. Clusters against demographic variables Survey A.docx

Table S3. Clusters against demographic variables Survey B.docx

Table S5. Survey B Clusters.docx

Table S4. Survey A Clusters.docx

## Data Availability

The data from this study are the property of Defence Research and Development Canada; public access is restricted.
