# Supplementary material for: Optimizing military mental health and stress resilience training through the lens of trainee preferences: A conjoint analysis approach
Source: Mil Psychol. 2024 Mar 14;37(3):175–86. doi: 10.1080/08995605.2024.2324647 (PMC12026029; doi:10.1080/08995605.2024.2324647)
Supplement: Table S1. Average Utility Scores of Survey A and Survey B.docx [file HMLP_A_2324647_SM0511.docx]

**Table S1.** Average Utility Scores of Survey A and Survey B.

| Survey A Attributes | *Utility Scores* | Survey B Attributes | *Utility Scores* |
| --- | --- | --- | --- |
| Skill Practice |  | Supplemental Content |  |
| without opportunity to practice skills | -1.44 | handouts and email | -0.85 |
| with opportunity to practice skills without simulated stress | 0.40 | a digital portal only accessible on DND network or devices | -0.41 |
| with opportunity to practice skills under simulated stress | 1.04 | a digital portal accessible from anywhere and on personal devices | 1.26 |
| Instructor Type |  | Leadership Buy-In |  |
| uniformed leaders | -1.18 | do not actively support/model training | -2.30 |
| uniformed peers | -0.84 | promote training | 0.62 |
| civilian mental health and resilience experts | 0.93 | model skills outside of R2MR context | 0.68 |
| uniformed mental health and resilience experts | 1.09 | provide opportunities to practice R2MR skills | 1.0 |
| Content Relevance/Applicability |  | Content Relevance/Applicability |  |
| never throughout the training | -1.79 | never throughout the training | -1.98 |
| throughout some of the training | 0.29 | throughout some of the training | 1.14 |
| throughout most of the training | 0.65 | throughout most of the training | 0.69 |
| throughout all of the training | 0.84 | throughout all of the training | 1.15 |
| Demographic Similarity Trainee/Trainer |  | Nudging |  |
| none of the time | -0.44 | a personalized app tailored to your performance and well-being | 0.89 |
| some of the time | 0.25 | generic emails sent through app or email | -0.40 |
| most of the time | 0.25 | posters mounted in common areas | -0.50 |
| all of the time | -0.06 |  |  |

***Note.*** DND = Department of National Defence. R2MR = Road to Mental Readiness.
