# Supplementary material for: Optimizing military mental health and stress resilience training through the lens of trainee preferences: A conjoint analysis approach
Source: Mil Psychol. 2024 Mar 14;37(3):175–86. doi: 10.1080/08995605.2024.2324647 (PMC12026029; doi:10.1080/08995605.2024.2324647)
Supplement: Table S2. Clusters against demographic variables Survey A.docx [file HMLP_A_2324647_SM0510.docx]

**Table S2.** Clusters against demographic variables Survey A.

|  |  | *n* (%) | | | |
| --- | --- | --- | --- | --- | --- |
| Variable |  | Combined  (*n* = 203) | Cluster 1  (*n* = 46) | Cluster 2  (*n* = 68) | Cluster 3  (*n* = 89) |
| Gender |  |  |  |  |  |
| Man |  | 160 (79.2) | 33 (71.7) | 53 (77.9) | 74 (84.1) |
| Woman |  | 42 (20.8) | 13 (28.3) | 15 (22.1) | 14 (15.9) |
| Age |  |  |  |  |  |
| Younger than 25 |  | 17 (8.4) | 3 (6.5) | 6 (8.8) | 8 (9.0) |
| 25 to 34 |  | 63 (31.0) | 7 (15.2) | 26 (38.2) | 30 (33.7) |
| 35 to 44 |  | 65 (32.0) | 19 (41.3) | 21 (30.9) | 25 (28.1) |
| 45 to 54 |  | 50 (24.6) | 15 (32.6) | 12 (17.6) | 23 (25.8) |
| Older than 55 |  | 8 (3.9) | 2 (4.3) | 3 (4.4) | 3 (3.4) |
| Military component |  |  |  |  |  |
| Regular Force |  | 175 (86.2) | 41 (89.1) | 61 (89.7) | 73 (82.0) |
| Reserve Force |  | 28 (13.8) | 5 (10.9) | 7 (10.3) | 16 (18.0) |
| Military element |  |  |  |  |  |
| Navy |  | 33 (16.3) | 2 (4.3) | 10 (14.7) | 21 (23.6) |
| Army |  | 113 (55.7) | 32 (69.6) | 34 (50.0) | 47 (52.8) |
| Air Force |  | 57 (28.1) | 12 (26.1) | 24 (35.3) | 21 (23.6) |
| Military rank |  |  |  |  |  |
| Junior NCM |  | 78 (38.4) | 14 (30.4) | 31 (45.6) | 33 (37.1) |
| Senior NCM |  | 49 (24.1) | 13 (28.3) | 15 (22.1) | 21 (23.6) |
| Junior Officer |  | 48 (23.6) | 13 (28.3) | 14 (20.6) | 21 (23.6) |
| Senior Officer |  | 28 (13.8) | 6 (13.0) | 8 (11.8) | 14 (15.7) |
| Professional mental health background |  |  |  |  |  |
| No |  | 160 (82.1) | 34 (79.1) | 56 (87.5) | 70 (79.5) |
| Yes |  | 35 (17.9) | 9 (20.9) | 8 (12.5) | 18 (20.5) |
| Experienced mental health problems in past^1^ |  |  |  |  |  |
| No |  | 20 (10.3) | 4 (9.3) | 5 (7.8) | 11 (12.5) |
| Yes |  | 171 (87.7) | 37 (86.0) | 58 (90.6) | 76 (86.4) |
| Prefer not to say |  | 4 (2.1) | 2 (4.7) | 1 (1.6) | 1 (1.1) |
| MHSR received |  |  |  |  |  |
| No |  | 72 (36.9) | 15 (34.9) | 29 (45.3) | 28 (31.8) |
| Yes |  | 123 (63.1) | 28 (65.1) | 35 (54.7) | 60 (68.2) |
|  |  | Mean (SD) | | | |
| Health locus of control |  |  |  |  |  |
| Score |  | 53.1 (11.2) | 51.2 (9.5) | 56.9 (12.0) | 51.3 (10.7) |

*Note.* NCM = non-commissioned member. MHSR = mental health and stress resilience.
^1^ “experienced a problem related to stress, emotions, alcohol, or anything else affecting their well-being”
