# Supplementary material for: Optimizing military mental health and stress resilience training through the lens of trainee preferences: A conjoint analysis approach
Source: Mil Psychol. 2024 Mar 14;37(3):175–86. doi: 10.1080/08995605.2024.2324647 (PMC12026029; doi:10.1080/08995605.2024.2324647)
Supplement: Table S3. Clusters against demographic variables Survey B.docx [file HMLP_A_2324647_SM0509.docx]

**Table S3.** *Clusters against demographic variables Survey B.*

|  |  | *n* (%) | | |
| --- | --- | --- | --- | --- |
| Variable |  | Combined  (n = 215) | Cluster 1  (n = 149) | Cluster 2  (n = 66) |
| Gender |  |  |  |  |
| Man |  | 156 (75.0) | 106 (71.6) | 50 (83.3) |
| Woman |  | 52 (25.0) | 42 (28.4) | 10 (16.7) |
| Age |  |  |  |  |
| Younger than 25 |  | 14 (6.6) | 9 (6.0) | 5 (7.9) |
| 25 to 34 |  | 55 (25.9) | 37 (24.8) | 18 (28.6) |
| 35 to 44 |  | 68 (32.1) | 49 (32.9) | 19 (30.2) |
| 45 to 54 |  | 63 (29.7) | 46 (30.9) | 17 (27.0) |
| Older than 55 |  | 12 (5.7) | 8 (5.4) | 4 (6.3) |
| Military component |  |  |  |  |
| Regular Force |  | 183 (86.3) | 129 (87.2) | 54 (84.4) |
| Reserve Force |  | 29 (13.7) | 19 (12.8) | 10 (15.6) |
| Military element |  |  |  |  |
| Navy |  | 33 (15.5) | 21 (14.1) | 12 (18.8) |
| Army |  | 104 (48.8) | 74 (49.7) | 30 (46.9) |
| Air Force |  | 75 (35.2) | 54 (36.2) | 21 (32.8) |
| Military rank |  |  |  |  |
| Junior NCM |  | 84 (39.6) | 59 (39.9) | 25 (39.1) |
| Senior NCM |  | 53 (25.0) | 33 (22.3) | 20 (31.2) |
| Junior Officer |  | 38 (17.9) | 29 (19.6) | 9 (14.1) |
| Senior Officer |  | 37 (17.5) | 27 (18.2) | 10 (15.6) |
| Professional mental health background |  |  |  |  |
| No |  | 161 (79.3) | 115 (79.9) | 46 (78.0) |
| Yes |  | 42 (20.7) | 29 (20.1) | 13 (22.0) |
| Experienced mental health problems in past^1^ |  |  |  |  |
| No |  | 31 (15.2) | 19 (13.2) | 12 (20.0) |
| Yes |  | 160 (78.4) | 119 (82.6) | 41 (68.3) |
| Prefer not to say |  | 13 (6.4) | 6 (4.2) | 7 (11.7) |
| MHSR received |  |  |  |  |
| No |  | 68 (33.3) | 47 (32.6) | 21 (35.0) |
| Yes |  | 136 (66.7) | 97 (67.4) | 39 (65.0) |
|  |  | Mean (SD) | | |
| Health locus of control |  |  |  |  |
| Score |  | 52.3 (10.1) | 52.0 (9.5) | 53.0 (11.5) |

*Note.* NCM = non-commissioned member. MHSR = mental health and stress resilience.
^1^“experienced a problem related to stress, emotions, alcohol, or anything else affecting their well-being”
