# Supplementary material for: Optimizing military mental health and stress resilience training through the lens of trainee preferences: A conjoint analysis approach
Source: Mil Psychol. 2024 Mar 14;37(3):175–86. doi: 10.1080/08995605.2024.2324647 (PMC12026029; doi:10.1080/08995605.2024.2324647)
Supplement: Table S5. Survey B Clusters.docx [file HMLP_A_2324647_SM0508.docx]

**Table S5**. *Survey B Clusters: k-mean Mean (SD) & Utility Scores*

|  | Cluster 1 | | Cluster 2 | |
| --- | --- | --- | --- | --- |
| Attribute | *M* (*SD*) | Utility Scores | *M* (*SD*) | Utility Scores |
| Supplemental Content |  |  |  |  |
| handouts and email | -1.26 (0.67) | -1.26 | 0.07 (0.88) | 0.07 |
| a digital portal only accessible on DND network or devices | -0.28 (0.39) | -0.28 | -0.72 (0.46) | -0.72 |
| a digital portal accessible from anywhere and on personal devices | 1.53 (0.65) | 1.53 | 0.64 (0.81) | 0.64 |
| Leadership Buy-In |  |  |  |  |
| do not actively support/model training | -3.02 (0.78) | -3.02 | -0.69 (0.99) | -0.69 |
| promote training | 0.75 (0.41) | 0.75 | 0.33 (0.49) | 0.33 |
| model skills outside of R2MR context | 0.91 (0.54) | 0.91 | 0.17 (0.63) | 0.17 |
| provide opportunities to practice R2MR skills | 1.35 (0.45) | 1.35 | 0.19 (0.57) | 0.19 |
| Content Relevance/Applicability |  |  |  |  |
| never throughout the training | -2.42 (0.62) | -2.42 | -0.97 (0.95) | -0.97 |
| throughout some of the training | 0.17 (0.09) | 0.17 | 0.06 (0.14) | 0.06 |
| throughout most of the training | 0.82 (0.22) | 0.82 | 0.41 (0.29) | 0.41 |
| throughout all of the training | 1.43 (0.48) | 1.43 | 0.51 (0.73) | 0.51 |
| Nudging |  |  |  |  |
| a personalized app tailored to your performance and well-being | 1.07 (0.75) | 1.07 | 0.50 (0.86) | 0.49 |
| generic emails sent through app or email | -0.51 (0.34) | -0.51 | -0.13 (0.48) | -0.13 |
| posters mounted in common areas | -0.56 (0.55) | -0.56 | -0.37 (0.62) | -0.37 |

*Note.* DND = Department of National Defence. R2MR = Road to Mental Readiness.
