# Supplementary material for: Optimizing military mental health and stress resilience training through the lens of trainee preferences: A conjoint analysis approach
Source: Mil Psychol. 2024 Mar 14;37(3):175–86. doi: 10.1080/08995605.2024.2324647 (PMC12026029; doi:10.1080/08995605.2024.2324647)
Supplement: Table S4. Survey A Clusters.docx [file HMLP_A_2324647_SM0507.docx]

**Table S4**. *Survey A Clusters: k-means Mean (SD) & Utility Scores*

|  | Cluster 1 | | Cluster 2 | | Cluster 3 | |
| --- | --- | --- | --- | --- | --- | --- |
| Attribute | *M* (*SD*) | Utility Scores | *M* (*SD*) | Utility Scores | *M* (*SD*) | Utility Scores |
| Skill Practice |  |  |  |  |  |  |
| without opportunity to practice skills | -0.64 (1.04) | -2.35 | -0.77 (0.86) | -0.77 | -2.37 (0.56) | -0.64 |
| with opportunity to practice skills without simulated stress | 0.45 (0.55) | 0.34 | 0.44 (0.43) | 0.46 | 0.35 (0.46) | 0.45 |
| with opportunity to practice skills under simulated stress | 0.19 (1.08) | 2.01 | 0.32 (0.94) | 0.31 | 2.02 (0.69) | 0.19 |
| Instructor Type |  |  |  |  |  |  |
| uniformed leaders | -0.01 (0.95) | -0.95 | -2.29 (0.73) | -2.31 | -0.94 (0.76) | -0.01 |
| uniformed peers | -0.09 (0.76) | -0.89 | -1.28 (0.62) | -1.29 | -0.89 (0.56) | -0.09 |
| civilian mental health and resilience experts | -0.93 (0.93) | 0.78 | 2.38 (0.70) | 2.41 | 0.78 (0.88) | -0.93 |
| uniformed mental health and resilience experts | 1.03 (0.30) | 1.06 | 1.19 (0.28) | 1.18 | 1.06 (0.27) | 1.03 |
| Content Relevance/Applicability |  |  |  |  |  |  |
| never throughout the training | -1.64 (0.62) | -2.11 | -1.45 (0.48) | -1.45 | -2.12 (0.41) | -1.64 |
| throughout some of the training | 0.31 (0.17) | 0.35 | 0.21 (0.13) | 0.21 | 0.35 (0.12) | 0.31 |
| throughout most of the training | 0.60 (0.13) | 0.73 | 0.58 (0.12) | 0.58 | 0.73 (0.11) | 0.60 |
| throughout all of the training | 0.73 (0.44) | 1.03 | 0.66 (0.36) | 0.66 | 1.03 (0.31) | 0.73 |
| Demographic Similarity Trainee/Trainer |  |  |  |  |  |  |
| none of the time | -0.59 (0.47) | -0.45 | -0.31 (0.45) | -0.32 | -0.46 (0.46) | -0.59 |
| some of the time | 0.33 (0.36) | 0.21 | 0.26 (0.35) | 0.26 | 0.21 (0.32) | 0.33 |
| most of the time | 0.29 (0.31) | 0.28 | 0.17 (0.31) | 0.18 | 0.28 (0.31) | 0.29 |
| all of the time | -0.03 (0.08) | -0.04 | -0.12 (0.06) | -0.12 | -0.04 (0.07) | -0.03 |
